# Trial for Malpractice, and One Thousand Dollars Damages

**Published:** 1847-08

**Authors:** 


ART. II. — *Trial for Malpractice, and One Thousand Dollars damages.*

*Francis Bugard* vs. *George Gross.* This was an action for alledged malpractice, tried in the Court of Common Pleas, at Buffalo, County of Erie, and State of New-York, June 21, 1847.

Judge Stevens presiding. I. T. Williams and Seth C. Hawley, counsel for plantiff; James Mullett and W. W. Peacock, counsel for defendant.

It appeared in evidence on the trial, that the plaintiff, 23 years old, is a steady, industrious farmer, residing on Eleven Mile Creek, in the town of Amherst; and that the defendant is a Botanic and Root-Doctor, practising in Williamsville, not far from Eleven Mile Creek.

On the 17th of September last, about 9 months since, Francis was chopping a log in the woods, and by the sudden displacement of that portion upon which he was standing, he was thrown backwards, striking upon the corner and back part of the humerus. Two or three hours afterwards he was seen by the defendant, who upon examination said it was out of joint, and perhaps broken; and after pouring warm water on it about ten minutes to reduce the swelling, he employed extension and counter-extension in a straight line, and professed to have reduced it, yet the arm now, as before, could not be flexed, except very slightly. Defendant then applied two shingle-splints, one to the arm, extending from the outer condyle of the humerus, upwards about three inches, and the other from the coronoid process downwards on the fórarm, about the same distance—for what purpose these splints were applied was never attempted to be shown. The arm was then suspended by the side of the body in n early straight line, and secured by a couple of handkerchiefs.

*September* 18.—Defendant took the splint off, and witnesses noticed that the bones were not in place. Defendant said it was "swelled up so bad he could hardly tell if it was right or not." The same dressings were re-applied and the arm placed in the same extended position—the only one in which it could be kept. Defendant also gave Francis a liniment to reduce the swelling, which "burnt like fire," and produced excoriations. Defendant then said "put warm water in a bottle, and put by the arm, may be he catched cold the night before, because he suffered so much." Said he once set an ankle which had been out six times, &c.

*September* 19.—Defendant examined it again and said it was not right—that Francis had got it out of place, (although it did not appear but that he had been extremely careful). Having again poured warm water on it during five minutes, to reduce the swelling, and make it yield better, two men were directed to make extension and counter-extension in a straight line, while defendant manipulated at the elbow. After about one minutes' pulling, Gross said he guessed it was in, and, the men ceased pulling. The arm, however, remained as before, nearly straight, and the deformity at the elbow continued.

The same splints and liniment were re-applied, with farther directions to pour milk under the dressings if the liniment smarted too much. When defendant went out he said "they generally have an iron box to set the elbow in, but I have not got one myself, I will get one of Dr. Ham." &c.

*September* 22.—Defendant removed dressings and tried to bend the arm, but could not. Saw an ecchymosis in axilla, and directed fomentations of wormwood and vinegar to be applied. The same splints were re-applied, and the liniment continued. Gross said it was "gaining very fast"—"it was right."

This treatment was continued 18 days, and the arm was then left in its present shape and position. About three or four weeks after the occurrence of the accident, Francis called on Gross; Gross proposed to "break it over again," and sent him to Buffalo for the opinions of Drs. Hill and Davis, both Thompsonians.

The testimony as to the facts having closed, Drs. Hamilton and Sprague were examined as to their professional opinions, &c.

Dr. Hamilton testified that he had seen the arm; that it is a backward luxation of radius and ulna; arm is nearly straight and admits of but very slight motion at elbow-joint; thinks it was always the same luxation, and without fracture; thinks the luxation could have been easily diagnosed within two or three hours after the accident. The swelling could not have been great at that time, or it would have obliterated the fossa between the olecranon and the inner condyle, which one of the witnesses swears was as manifest then as now; if a fracture of any of the bones about the point had actually existed, it would be apparent now, since the treatment was not such as would be proper for any fracture about the elbow-joint, and a deformity at the point of fracture must have resulted. The reduction of this luxation at an early hour, is easily effected—always bends the forarm upon the arm to displace the coronoid process from the fossa of the humerus. This is the practice of all the modern surgeons except Liston. Liston straightens the arm, but carries it far back, so as to completely relax the triceps. The plan described by the witnesses as pursued by the defendant was not the same; he pulled in a straight line aud directly forwards, which put the triceps upon the stretch. It would require very great power to reduce the arm in this way. If it were reduced it could have been bent up to at least a right angle. This is the position in which it ought to have been placed—no splints were necessary. We sometimes use a moveable right-angled splint, when it is a child, who will tumble about, but the splints employed could not have been of the least service. The application of stimulating liniments was bad treatment. The reduction could not have been as easily effected on the third as on the first day; if it be true that fractures can be as well reduced after a few days as at first, it certainly is not so with dislocations—every day increases the difficulty. Dislocated elbow, radius and ulna backwards, have been reduced after several weeks, but generally the witness would be unwilling to make the attempt after six weeks. There is danger to the limb when the attempt is made at two weeks, and witness would so state to the patient before attempting the reduction.

Dr. Sprague testified (Dr. Sprague was requested by the counsel for plaintiff to examine the arm, which he did) that he had examined the arm, it is deformed; there is a dislocation of the radius and ulna backwards; bones are situated in this manner (showing the position of the bones upon a skeleton of an arm which was handed to him) the coronoid process is lodged in the fossa of the humerus. Witness reduces this dislocation by bending the arm forcibly across the knee or around the back of a chair, and then he sometimes suddenly brings it straight. The course pursued by the defendant would not be likely to reduce the bones; the splints used in this case could not have been of any service; the arm ought to have been kept at right angles; generally no great difficulty in discovering this dislocation; not very liable to get out of place; would not generally attempt to reduce a dislocation of this kind after five, six, or seven weeks,

Dr. Milo W. Hill, (a Botanic physician of Buffalo) testified on the part of the *defence*, that plaintiff came into his office in February; said he came to have his arm examined; defendant came with him; wished to know what could be done with it. Dr. Hill examined, he then took him into a Dr. Field's office (an oculist). Dr. Hill said he was formerly of the old school, but, is now a Botanic doctor. Dr. H., advised him to go to some other doctor, Dr. Sprague he thinks.

Dr. Wm. Field testified that he had heard Dr. Hill, and concurred in all his statements.

Dr. Davis, (Botanic Doctor), testified only in relation to the mental condition of one of the defendant's witnesses.

*The Jury returned a verdict for the plaintiff, of one thousand dollars.*

